# UrduSER: A comprehensive dataset for speech emotion recognition in Urdu language

**DOI:** 10.1016/j.dib.2025.111627

**Published:** 2025-05-08

**Authors:** Muhammad Zaheer Akhtar, Rashid Jahangir, QuratUl Ain, Muhammad Asif Nauman, Mueen Uddin, Syed Sajid Ullah

**Affiliations:** a Department of Information Technology, The Islamia University of Bahawalpur, Bahawalpur 63100, Pakistan; b Department of Computer Science, COMSATS University Islamabad, Vehari Campus 61100, Pakistan; c Riphah School of Computing & Innovation, Riphah International University, Lahore, Pakistan; d College of Computing and IT, University of Doha for Science and Technology 24449, Qatar; e Department of Information and Communication Technology, University of Agder (UiA), N¬4898 Grimstad, Norway

**Keywords:** Speech emotion recognition, Signal processing, Deep learning, Urdu language, Dataset

## Abstract

Speech Emotion Recognition (SER) is a rapidly evolving field of research that aims to identify and categorize emotional states through speech signal analysis. As SER holds considerable socio¬cultural and business significance, researchers are increasingly exploring machine learning and deep learning techniques to advance this technology. A well-suited dataset is a crucial resource for SER studies in a specific language. However, despite being the 10th most spoken language globally, Urdu lacks SER datasets, creating a significant research gap. The available Urdu SER datasets are insufficient due to their limited scope, including a narrow range of emotions, small datasets, and a limited number of dialogs, which restricts their usability in real-world scenarios. To fill the gap in existing Urdu speech datasets, an Urdu Speech Emotion Recognition Dataset (UrduSER) is developed. This comprehensive dataset consists of 3500 speech signals from 10 professional actors, with a balanced mix of males and females, and diverse age ranges. The speech signals were sourced from a vast collection of Pakistani Urdu drama serials and telefilms available on YouTube. Seven emotional states are covered in the dataset: Angry, Fear, Boredom, Disgust, Happy, Neutral, and Sad. A notable strength of this dataset is the diversity of the dialogs, with each utterance containing almost unique content, in contrast to existing datasets that often feature repetitive samples of predefined dialogs spoken by research volunteers in a laboratory environment. To ensure balance and symmetry, the dataset consists of 500 samples for each emotional class, with 50 samples per actor per emotion. An accompanying Excel file provides a detailed metadata index for each audio sample, including file name, duration, format, sample rate, actor information, emotional state, and the Urdu dialogue script. This comprehensive metadata index enables researchers and developers to efficiently access, organize, and utilize the UrduSER dataset. The UrduSER dataset underwent a rigorous validation process, integrating expert validation to confirm its validity, reliability, and overall suitability for research and development purposes.

Specifications Table

**Table:** 

Subject	*Signal Processing*
Specific subject area	*Speech analysis, Speech emotion recognition, Voice Recognition, Audio Sentiment Analysis*
Type of data	*Audio*
Data collection	*Urdu voice is captured from different serials of professional actors. The audio files are captured in seven different emotions (Anger, Boredom, Disgust, Fear, Happy, Neutral, Sad) using 3500 Urdu sentences. Some examples of sentences are listed below:* (Sad): (Neutral): (Happy): (Disgust): (Boredom): (Fear): (Anger):
Data source location	*City: Vehari* *Country: Pakistan* *Longitude: 30.031164566772617* *Latitude: 72.3140089586199*
Data accessibility	Repository name: Mendeley Data Data identification number: 10.17632/jcpfjnk5c2.4 Direct URL to data: https://data.mendeley.com/datasets/jcpfjnk5c2/4
Related research article	*None*

## 1 Value of the Data

Novel SER systems have expedited the development of empathetic machines, human-machine interaction and improved user experiences. However, the foundation of intelligent AI systems pivots the availability of high-quality, emotionally rich language specific datasets. Despite Urdu being one of the most widely spoken and understood languages globally, the existing Urdu speech datasets for emotion detection are scarce and plagued by limitations. These datasets often feature a restricted number of dialogues recorded in controlled studio settings by non-professional actors, inadequate instances of utterances for robust model learning, and a lack of emotional annotations in spontaneous conversation repositories, rendering them unsuitable for SER models. To address this gap, an Urdu speech emotion recognition dataset (UrduSER), a realistic and comprehensive dataset is designed to advance Urdu SER systems and human-machine interaction research, ultimately contributing to the development of more sophisticated and empathetic AI systems. The proposed dataset has the followings values:

• UrduSER dataset can be effectively employed to train artificial intelligence models for SER tasks with robust generalization.
• It offers valuable support for researchers to explore emotional expression in the Urdu language across various research domains, including audio surveillance, clinical studies, and home automation.
• The dataset is well-balanced, ensuring equal representation of speech-audio recordings across different emotional categories, with an equal number of male and female participants from a diverse age range.
• Researchers can analyze various audio features, such as time and frequency-based attributes, from this dataset and apply them to different artificial intelligence models to enhance SER performance in the low-resource Urdu language context.

## 2 Background

The recognition of emotions from speech signals, also known as Speech Emotion Recognition (SER), has been a rapidly evolving field of research in recent years. SER systems aim to identify and classify human emotions from speech patterns, enabling a wide range of applications in areas such as human-computer interaction, affective computing, and healthcare [1,2]. This growing interest in SER research has led to the development of various SER systems for different languages, including English, Spanish, and Mandarin [3]. Recent studies have also explored the use of deep learning techniques, such as convolutional neural networks (CNNs), recurrent neural networks (RNNs), and hybrid models for SER tasks [4]. For instance, [5] developed a hybrid model using CNN and BiLSTM with several attention mechanisms. The authors utilized two types of features namely Mel Frequency Cepstral Coefficients (MFCCs) and Mel-spectrograms as input to the hybrid CNN-BiLSTM model. The attention mechanism helped to focus on energy, frequency, and time variations in Mel-spectrograms related to emotion. While MFCCs and its derivatives were used to identify the dynamic sequential variations. The attention-extracted information from CNN and BiLSTM models was merged and fed to the deep neural network. The model was evaluated on three datasets: IEMOCAP, Emo-DB, and a private Amritaemo_Arabic dataset. The model not only achieved excellent results on self-constructed Arabic datasets, but also on two widely used benchmark datasets for SER. Furthermore, researchers have emphasized the importance of considering multimodal cues, such as speech, text, and visual features, for improving the accuracy of SER systems [6,7]. A crucial aspect of developing accurate and robust SER systems is the availability of reliable datasets. High-quality datasets like Emo-DB and IEMOCAP provide a solid foundation for training and testing SER models, enabling the development of systems that can effectively recognize and classify human emotions from speech patterns [3].

The existing benchmark datasets are collected in European languages such as German (EmoDB) and English (IEMOCAP) while Urdu introduce a South Asian perspective in the field of SER. The emotions expressed in voice recorded in Urdu differ significantly from Western languages due to linguistic structures and cultural contexts. The linguistic structures define the rules and patterns inside a language like grammar, vocabulary, sentence construction and expression of emotions. For instance, Urdu has many words (``'' vs ``'' for ``you'') that can alter the emotional tone. Secondly, cultural contexts refer to the social and cultural ``rules'' about expressing emotions. Therefore, there is a significant gap in the availability of comprehensive resources for languages such as Urdu to fill this major linguistic and cultural gap. Urdu is the national language of Pakistan and the 10th most widely spoken language globally, with over 230 million speakers. Unfortunately, Urdu lacks comprehensive resources in the domain of speech emotion recognition, and its significance has not been adequately reflected in affective computing and speech emotion analysis research. This article develops an Urdu speech emotion corpus to bridge the existing gap, enabling emotion recognition systems for applications in human computer interaction (HCI), sentiment analysis, and mental health support. The proposed corpora will facilitate the research of emotional expressions in Urdu speech, paving the way for future research in this vital area. Although Urdu speech emotion datasets are available, they still face a significant limitation in effectively capturing real-time emotional expressions. This shortcoming can be attributed to the prevalent use of studio recordings, restricted dialogue ranges, and the employment of non-professional actors. Consequently, a substantial disparity exists between the datasets and genuine emotional speech, underscoring the necessity for more extensive and realistic datasets to effectively represent the intricacies of emotional expression in Urdu.

**Fig. 1:**
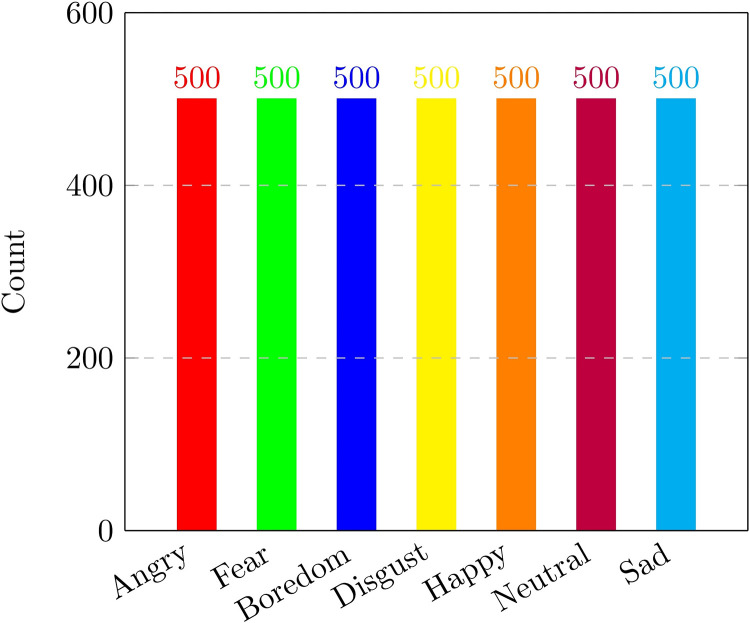
Emotion-wise speech recording distribution in the UrduSER dataset.

This article introduces the UrduSER dataset, comprising emotional speech segments sourced from Pakistani dramas, telefilms, and reality shows. Characterized by their captivating narratives, strong acting, and realistic portrayals, Pakistani dramas have achieved international recognition. The USEC dataset's value lies in its diverse, non-repetitive, and spontaneous dialogue, providing a more realistic and robust resource for advancing SER research and benchmarking. The UrduSER dataset embraces audio speech recordings of seven fundamental emotions, facilitating a seven-class classification problem with real-world applicability as given in Table 1. The dataset features speech from 10 actors spanning ages 20–70, acquainted with age-related variability. Furthermore, the dataset is carefully balanced (Fig. 1), with an equal distribution of male and female speech samples across each emotion category, enhancing its reliability and generalizability.

**Table 1:** Emotion-wise description of UrduSER dataset.

## 3 Data Description

SER explicitly deals with language specific research that identifies and classifies speech signals to determine emotional states. However, appropriate datasets are vigorously important for SER studies when it comes to emotion detection for a specific language. The developed UrduSER is lifelike Urdu speech emotion dataset encompassing seven emotional states extracted from realistic emotional scenes picked from Pakistani Dramas, telefilms, and reality shows.

**Fig. 2:**
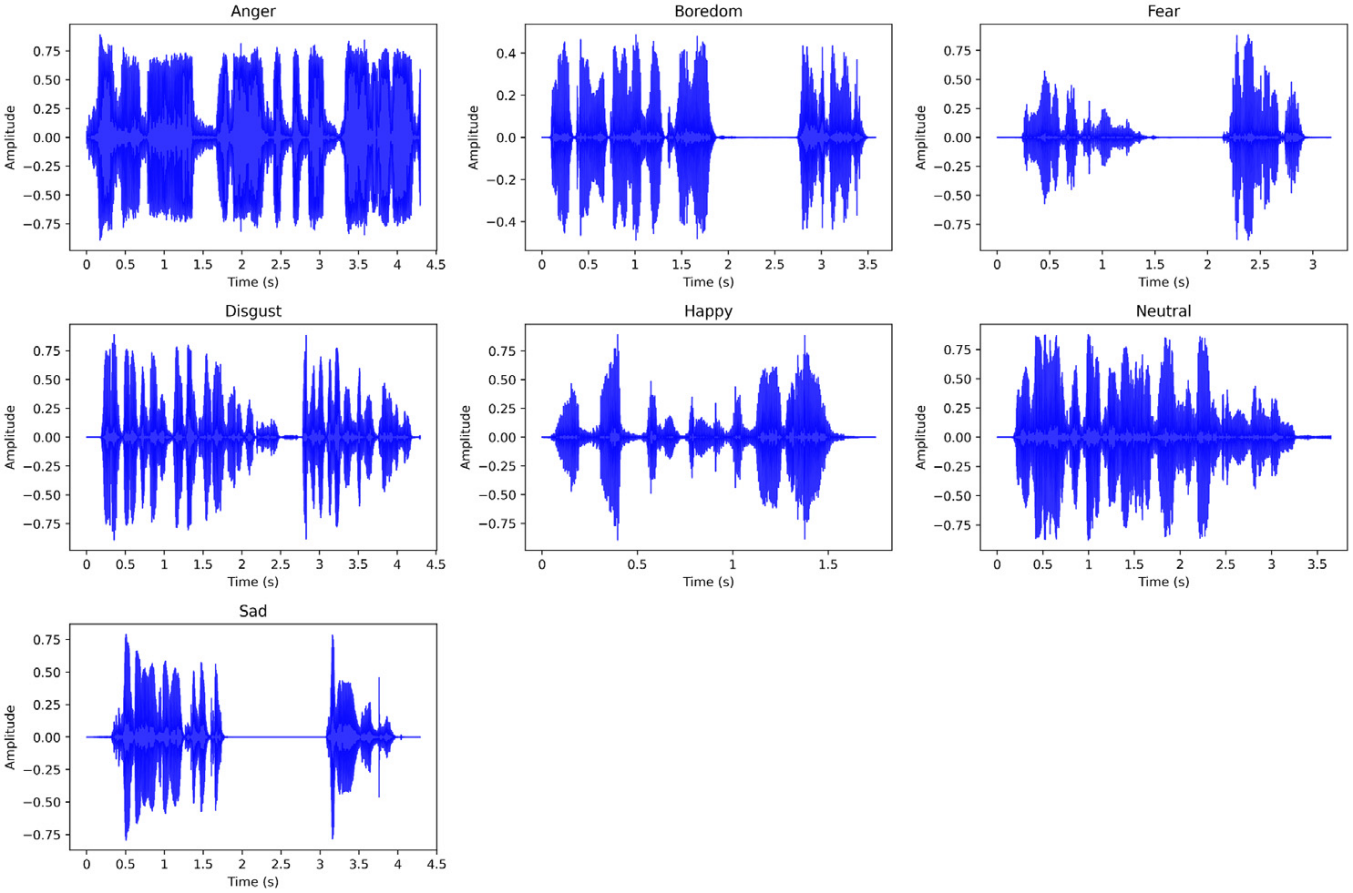
Sample waveform of a randomly selected audio of each emotion, (a) anger, (b) boredom, (c) disgust, (d) fear, (e) happy, (f) neutral and (g) sad emotions of UrduSER.

The dataset comprises seven distinct speech emotions including anger, anxiety/fear, boredom, disgust, happiness, neutral, and sad as shown in Fig. 2. Due to the similar acoustic characteristics and linguistic features of fear and anxiety, dialogues expressing these emotions have been consolidated into a single category. Each emotional category is represented by a separate folder containing 500 audio samples, resulting in a total of 3500 files across seven folders. This unified dataset is characterized by a consistent file format, with each audio sample stored in standard WAV format, featuring a sample rate of 44,100 Hz, bit rate of 1411 Kbps, and bit depth of 32-bit float.

The UrduSER dataset comprises speech clips collected from ten professional actors, renowned in the drama industry, with a balanced gender distribution of five male and five female actors. Each actor contributed 350 audio samples, with 50 samples representing each of the seven emotions (anger, anxiety/fear, boredom, disgust, happiness, neutral, and sadness). Diverse sources were utilized to collect the 50 audio samples for each emotional state from a particular actor or actress, including multiple drama serials or telefilms. This approach was employed because actors often play distinct roles in different productions, resulting in varied performances. For example, when collecting angry emotion samples from actor Firdous Jamal, scenes were selected from at least three different drama serials. This deliberate sampling strategy ensures a rich and diverse dataset, showcasing a broad spectrum of emotional expressions from skilled performers.

Remarkable variability is a hallmark of this dataset, where individual dialogues exhibit unique traits for each emotional category and even for each actor. Notably, the dataset was not recorded in a laboratory setting for SER research purposes, unlike other available Urdu speech datasets with limited number of predefined dialogues. With nearly 3500 unique samples, the dataset offers an unparalleled level of diversity in speech patterns. An Excel file (link) has been created to document the dataset, providing detailed information for each audio sample. The file includes columns for audio file name, duration, file format, sample rate, actor identification number, actor name, actor gender, emotional state, and script of the dialogue written in Urdu language. This comprehensive index facilitates easy access and organization of the dataset's metadata, making it a valuable resource for researchers and developers working with the UrduSER dataset.

### 3.1 Comparison with existing urdu speech datasets

The performance and reliability of SER systems critically depend on the availability and quality of speech signal resources in various languages. This section presents a comprehensive review of existing Urdu speech datasets, followed by a comparative analysis with our proposed dataset, specifically designed to meet the requirements of SER applications. Table 2 summarizes formerly developed prominent Urdu speech datasets for emotion recognition, including our contribution.

**Table 2:** Overview of Urdu speech datasets for emotion recognition.

Language	URDU	Urdu-Sindhi	SEMOUR	UrduSER
Language	Urdu	Urdu+Sindhi	Urdu	Urdu
Publication Year	2018	2020	2021	2024
Emotions	4	7	8	7
Total Audios	400	1435	15,040	3500
Environment	Spontaneous	Acted	Acted	Realistic
Number of Dialogues	400	70	235	3500
Actors (M:F)	27M:11F	-	4M:4F	5M:5F
Data Source	TV Talk Shows	WhatsApp Audio	Radio Studio	YouTube
WAV file size	76 MB	589 MB	47MB	1.80 GB
Sample Rate	22kHz	48kHz	16kHz	44.1kHz
Uttered Emotions				A, B, D, F, H, N, S

** *A*= Anger, *B*=Boredom, *D*=Disgust, *F*=Fear, *H*=Happy, *N*=Neutral, *S*=Sad.

The development of Urdu SER systems is hindered by a paucity of resources. However, a notable contribution is the spontaneous URDU dataset presented by [8], featuring 400 utterances from 38 actors (with a gender imbalance) sourced from television talk shows. This dataset encompasses a restricted emotional range, covering only four primary emotions: anger, sadness, neutrality, and happiness. The dataset's limited size and gender imbalance, combined with the narrow emotional scope, highlights the urgent need for expanded and diverse resource creation to drive advancements in Urdu speech emotion recognition research. In a separate investigation, the authors [9] developed the Urdu-Sindhi Speech Emotion Corpus, a unique dataset comprising audio recordings of undergraduate students expressing seven distinct emotional states. Participants uttered 10 scripted sentences in both Urdu and Sindhi, resulting in a total of 1435 audio recordings collected via WhatsApp voice notes. Nevertheless, the authors failed to provide essential demographic details, including the number of participants and gender distribution.

The researchers [10] constructed a large-scale emotional speech corpus in Urdu named SEMOUR+, featuring 27,640 audio samples comprising 14 hours recording annotated with eight emotions. The script consisting of 235 instances (including single words, two-word phrases, and complete sentences), was recorded by 24 speakers (with balanced gender representation) in a radio studio. This repository offers a significant contribution to the field of SER, providing a substantial and diverse collection of emotional speech samples in Urdu. Interestingly, the author’s proposed model only achieved a modest average accuracy of 56 % in speaker-independent experiments, even when trained on the entire dataset. In a similar study [11] created an emotional speech corpus comprising 2500 audio clips, recorded in a university laboratory by 20 speakers (10 male, 10 female) expressing five emotions: anger, happiness, neutrality, disgust, and sadness. Each speaker uttered five common Urdu phrases five times per emotion, yielding 125 clips per speaker. After discarding 200 clips due to improper utterances during validation, the final corpus was compiled. However, the dataset's limited age range (20–40 years) and restricted script may detract from its representativeness and benchmark potential.

In a related study [12], an emotional Urdu speech corpus was developed to explore acoustic and prosodic correlations. The corpus consisted of 23 sentences spoken by four speakers, evoking four emotional states, recorded over four sessions of three hours each using a microphone. However, the authors failed to report the total number of instances used in their experiment, leaving this crucial detail unspecified. In contrast, the proposed UrduSER stands out as a more realistic and comprehensive Urdu speech emotion dataset, surpassing existing ones in several ways. It was carefully compiled from a diverse range of Pakistani dramas, telefilms, and reality shows, featuring esteemed actors from various age groups and equal gender representation. Each audio file contains a unique script, and the dataset encompasses a wide spectrum of emotions with 500 samples per emotion. Furthermore, a detailed Urdu text file accompanies the dataset, providing valuable metadata for all 3500 recordings, including file information, emotion type, actor details, and context, thereby paving the way for advanced sentiment analysis as well as SER in Urdu.

### 3.2 Comparison with benchmark speech emotion datasets

Given that the speech emotions comprise a complex array of input variables, evaluations of emotional responses must incorporate a requisite degree of naturalness to ensure that the resultant performance assessments approximate real-world scenarios with sufficient fidelity [13]. Standardized speech emotion databases have emerged in various languages, facilitating cross-linguistic and cross-cultural research in emotion recognition and affective computing. The SAVEE database [14] is a multimodal corpus featuring audio-visual recordings of 480 British English utterances spoken by four male actors (ages 27–31). The dataset is annotated with seven emotional categories (happiness, sadness, anger, fear, surprise, disgust, and neutral) and consists of phonetically balanced sentences. Recordings were made in a controlled environment with high-quality audio-visual equipment. The Berlin Database of Emotional Speech (EmoDB) [15] is widely used speech emotion recognition dataset recorded by gender balanced ten professional German language speakers. The datasets consists of total 535 utterances capturing seven emotions; anger, boredom, anxiety, happiness, sadness, disgust, and neutral. The authors constructed 10 sentences for recordings at sampling frequency of 48 kHz and later down sampled to 16 kHz. The IEMOCAP (Interactive Emotional Motion Capture) dataset [16] is a renowned multimodal emotional intelligence corpus, comprising approximately 12 hours of audio-visual recordings from 10 actors (5 males, 5 females). The dataset captures spontaneous and elicited emotions through scripted and improvised dialogues, including audio, facial expressions, body gestures, and head movements. Emotions are annotated into 10 categories (happiness, sadness, anger, fear, surprise, frustration, disappointment, neutral, excitement, and other). The Ryerson Audio-Visual Database of Emotional Speech and Song (RAVDESS) [17] is an English multimodal dataset consisting of 7352 audio and video recordings from 24 actors (12 males, 12 females), capturing emotional expressions through speech and song. The dataset encompasses eight emotions (neutral, calm, happy, sad, angry, fearful, disgust, and surprised) at two intensity levels (normal and exaggerated), providing a valuable resource for research in emotion recognition, affective computing, and human-computer interaction. A comparative analysis of UrduSER with benchmark speech emotion recognition datasets is given in Table 3.

**Table 3:** Overview of emotional speech datasets.

Dataset	Emotions	Language	Speakers	Setting	Files	Type
SAVEE	A, D, F, H, N, S, Sr,	British English	4	CVSSP's 3D vision laboratory	480	Acted
EmoDB	A, B, D, F, H, N, S	German	5 M: 5 F	Controlled studio environment	535	Acted
IEMOCAP	A, D, E, F, H, N, Sr	English	5 M: 5 F	Controlled studio environment	151 recording	Acted
RAVDESS	A, C, D, F, H, N, S, Sr	English	12 M: 12 F	controlled studio environment	7352	Acted

** *A*=Anger, *B*=Boredom, *C*=Calm, *D*=Disgust, *E*=Excited, *F*=Fear, *H*=Happy, *N*=Neutral, *S*=Sad, Sr=Surprise.

## 4 Experimental Design, Materials and Methods

**Fig. 3:**
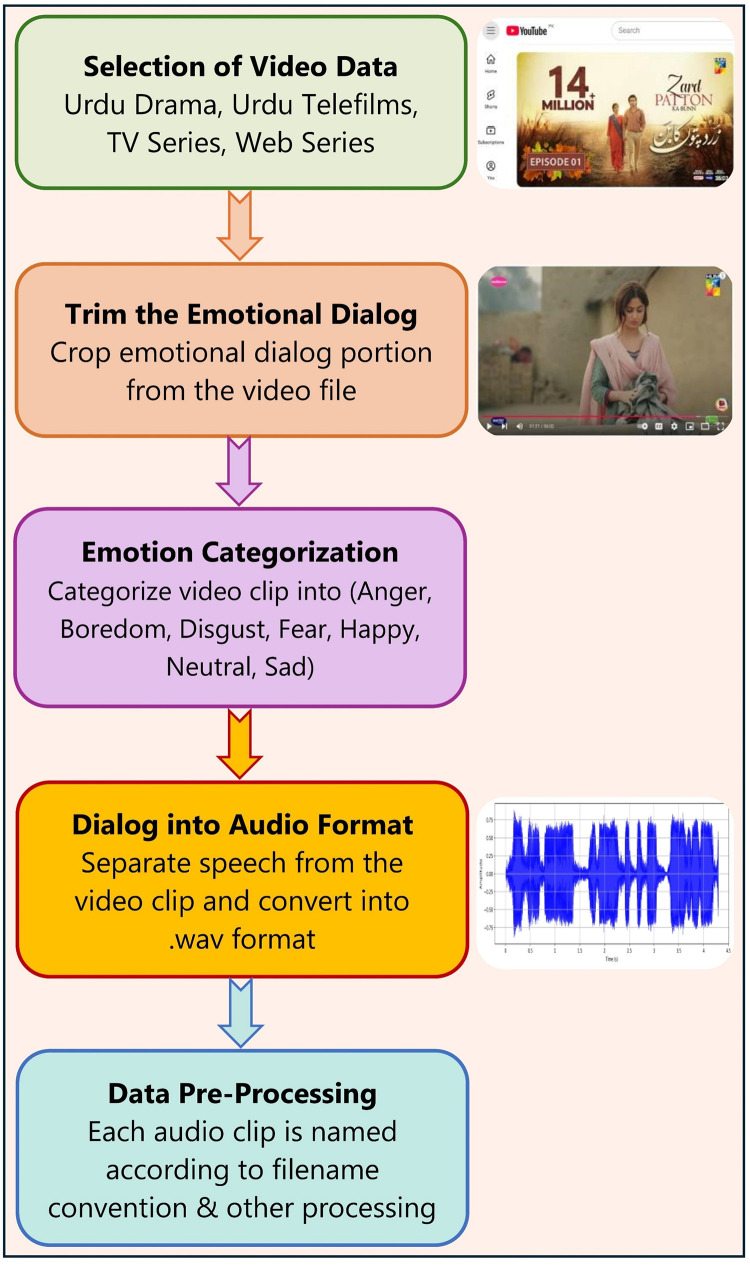
Pipeline diagram of the UrduSER preparation.

A five-stage process, as illustrated in Fig. 3, was utilized to construct the UrduSER. The following subsections provide a comprehensive overview of each stage.

### 4.1 Data collection

The emotional spectrum of UrduSER includes seven distinct categories: anger, fear, boredom, disgust, happiness, sadness, and neutrality, providing a detailed representation of human emotions.

### 4.2 Source selection

To create a culturally diverse and emotionally rich corpus, the proposed work leverages Pakistani drama serials, telefilms, and TV shows accessible on YouTube. A systematic two-stage selection process was employed to ensure the quality and relevance of the dataset, comprising the identification of renowned actors and the selection of pertinent scenes showcasing their performances.

### 4.3 Actor selection

A diverse pool of ten professional actors (five males, five females) was carefully curated for the UrduSER, taking into account several key factors. These factors included an age range of 20–65 years, regional diversity representing various Urdu-speaking regions, and acting versatility encompassing a range of dialects and emotions. Furthermore, the actors' ability to convey emotions across the entire spectrum, from neutrality to extremity, was a crucial consideration. This meticulous selection process ensures that the corpus boasts a rich and diverse emotional representation.

### 4.4 Scene selection

Following the actor selection process, a large collection of Pakistani Urdu drama serials and telefilms featuring these actors was compiled. Specific scenes from these dramas and telefilms were then carefully selected for each actor, corresponding to each emotion. The chosen scenes contain a range of emotional dialogues, covering the entire emotional spectrum. The UrduSER corpus, however, specifically focuses on seven basic emotions.

### 4.5 Audio extraction and emotional categorization

Each audio-visual scene comprised multiple dialogs, with each dialog delivered in a specific emotion. The selected scenes were manually analyzed, considering seven distinct emotions. To facilitate this process, the audio recording and editing application Audacity was employed to extract and categorize individual dialogs by emotion type. Throughout the dataset preparation, three native Urdu speakers (the authors) were directly involved. A rigorous verification process was implemented, wherein one author initially categorized each dialog by emotion, a second author independently verified the labeling, and a third author reviewed the entire process and randomly checked several samples to ensure quality and accuracy.

To capture the emotional nuances of each dialog, audio extraction was performed on a per-dialog basis, trimming specific portions of the video scene. After multiple viewings of each selected scene, audio extraction was manually conducted using Audacity 3.6.1, directly from YouTube. The extracted audio files have a sample rate of 44,100 Hz and are stored in WAV format. The duration of each file ranges from 2–6 s, depending on the emotion type and dialog utterance. A total of over 5000 audio extractions were trimmed, representing all seven emotions, with 3500 emotionally rich audio speech files selected for inclusion.

### 4.6 Data preprocessing

The preprocessing of UrduSER involved a three-step procedure conducted using Audacity 3.6.1 with the OpenVINO music separation and noise suppression extension. First, music separation was performed to isolate the vocal component from background music and other audio elements. Using Audacity’s OpenVINO tool, the ``Vocals'' track was selected and removed the ``Drum,'' ``Bass,'' and ``Other'' components. Second, noise suppression was applied using the OpenVINO noise suppression effect with default settings which allowed automatic removal of noise artifacts while preserving speech quality. Third, silence trimming was conducted by using Audacity’s standard trim audio function with a silence threshold of −60 dB to remove silent segments at the beginning and end of each clip. All steps were performed manually via Audacity’s graphical user interface, without writing any custom scripts.

### 4.7 Naming convention

Assigning a unique name to each file using a standardized naming convention. The naming convention, outlined in Table 4, comprises four components: actor number, gender, emotional state, and speech number. For instance, the filename “2_1_3_04.wav” refers to “AijazAslam_Male_Boredom_4th Speech”, and the filename “7_1_7_30.wav” refers to “IqraAziz_Female_Sad_30th Speech”.

**Table 4:** Audio file naming convention description.

Identifier	Description
Actor Number	01 = Mehmood Aslam, 02 = Aijaz Aslam, 03 = Firdous Jamal, 04 = Imran Ashraf, 05 = Nayyar Ejaz, 06 = Saba Qamar, 07 = Iqra Aziz, 08 = Ayeza Khan, 09 = Asma Abbas, 10 = Sajal Ali
Gender	0 = Male, 1 = Female
Emotional State	1 = Angry, 2 = Fear, 3 = Boredom, 4 = Disgust, 5 = Happy, 6 = Neutral, 7 = Sad
Speech Number	01 = 1st Speech, 02 = 2nd Speech, …, 50 = 50th Speech

### 4.8 Dataset organization

The UrduSER dataset is structured in a clear and organized manner, with a main folder containing ten subfolders, one for each actor. Each actor's folder has seven subfolders, one for each emotion, and each emotion folder contains 50 audio extractions. This results in a total of 3500 audio files. An accompanying Excel file provides detailed metadata for each track, including file name, duration, format, sample rate, sample type, actor name, gender, emotion, and context.

### 4.9 Dataset validation

**Fig. 4:**
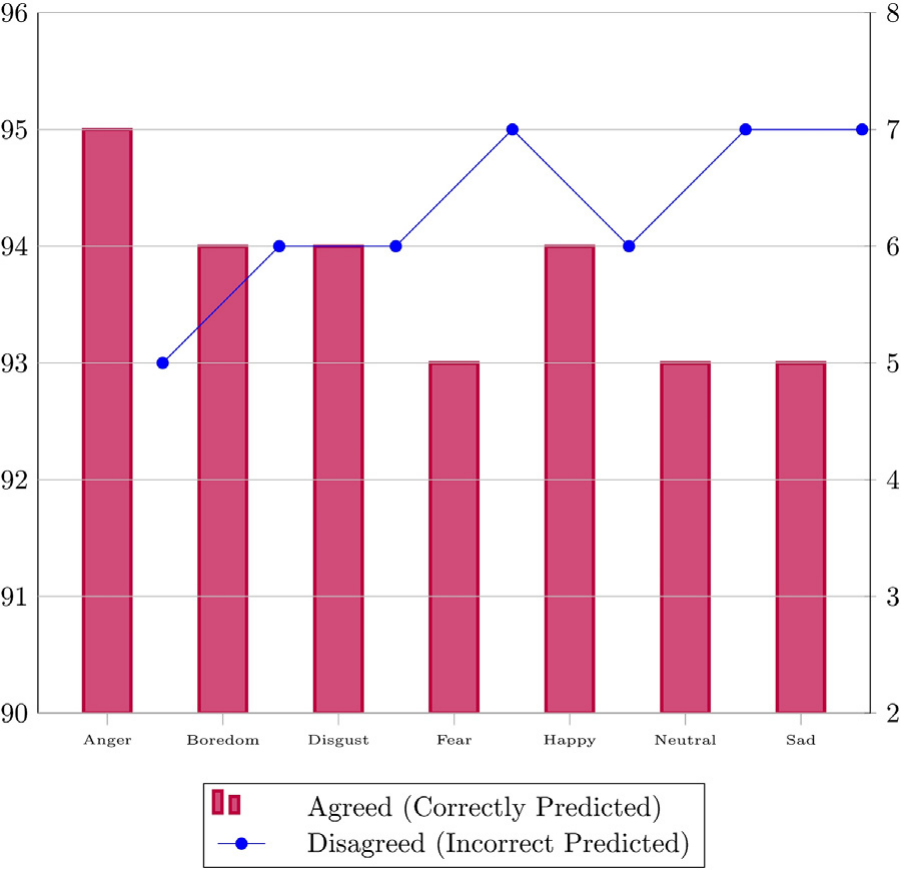
Statistics of data validated by experts.

The UrduSER dataset was subjected to a comprehensive validation process, incorporating expert validation to ensure its validity and reliability. Expert validation was conducted by designing a survey form and distributing it to native Urdu language speakers, specifically undergraduate university students. Participants were given 10 random audio samples from the dataset and asked to carefully listen to each file and select the emotion type that best represented the audio. The validation form designed for participants is given in Table 5. The results from approximately 100 participants were then compared to the original annotations, yielding an accuracy of 94 % as shown in Fig. 4.

**Table 5:** UrduSER validation form.

## Limitations

In future, spontaneous emotions collected from real-world sources such as interviews, phone conversations, and crowd-sourced recordings will be incorporated to expand the UrduSER dataset. This extension will address the limitation of acted speech and will create a more diverse dataset and enhance the applicability of UrduSER dataset in real-world tasks.

## Ethics Statement

This article utilized emotional speech dialogs from Pakistani drama serials, telefilms, and TV shows that were publicly available on YouTube. To mitigate copyright concerns, we carefully selected clips that were either explicitly licensed under Creative Commons or used short excerpts consistent with fair use principles for research and academic purposes. The dialogs were performed by professional actors for public audiences. Nevertheless, the dataset only includes the extracted speech audio, not the original video content, minimizing any potential copyright issues.

## Credit Author Statement

**Muhammad Zaheer Akhtar:** Methodology, Software, Formal analysis, Data curation; **Rashid Jahangir:** Conceptualization, Data curation, Software, Writing – original draft, Supervision, **Qurat Ul Ain:** Methodology, Supervision, Project administration, **Muhammad Asif Nauman:** Writing – review & editing, Software, Conceptualization, **Mueen Uddin:** Validation, Investigation, Writing – review & editing, **Syed Sajid Ullah:** Conceptualization, Writing – review & editing.

## Data Availability

Mendeley DataUrduSER: A Dataset for Urdu Speech Emotion Recognition (Original data).
